# The FtsH-Inactive Protein FtsHi5 Is Required for Chloroplast Development and Protein Accumulation in Chloroplasts at Low Ambient Temperature in Arabidopsis

**DOI:** 10.3389/fpls.2021.830390

**Published:** 2022-02-03

**Authors:** Jin-Yu Li, Jing-Liang Sun, Ying-Ying Tian, Jian-Xiang Liu

**Affiliations:** ^1^State Key Laboratory of Plant Physiology and Biochemistry, College of Life Sciences, Zhejiang University, Hangzhou, China; ^2^College of Environment and Resources, Dalian Nationalities University, Dalian, China

**Keywords:** low temperature, FtsHi5, proteostasis, temperature sensitive, chloroplast biogenesis

## Abstract

Chloroplasts are indispensable for higher plants. The growth and development of plants are very sensitive to environmental temperature changes, and chloroplast development is also regulated by adverse environmental temperatures. However, the molecular mechanism of how plants coordinate chloroplast development and environmental temperature changes remains largely unknown. Here, a temperature-conditioned chloroplast development defective mutant *thermo-sensitive mutant in leaf color 2* (*tsl2*) of *Arabidopsis* was obtained through a forward genetic screening. The *tsl2* mutant showed a weak yellowish phenotype at normal growth temperature (22°C), and the phenotype was more pronounced at low growth temperature (16°C) and largely rescued at high growth temperature (29°C). Bulk Segregant Analysis (BSA) revealed that *TSL2* encodes FtsH-Inactive Protein 5 (FtsHi5). Genetic complementation analysis confirmed that complemented expression of *FtsHi5* rescued the chlorophyll content and thylakoid development defects observed in *tsl2* mutants at 16°C. Quantitative mass spectrometry analysis with Tandem Mass Tag (TMT) isobaric labeling revealed broad changes in the chloroplast proteome of *tsl2* mutant plants at low temperature, which is agreed with the impaired chloroplast biogenesis and function in *tsl2* plants. Together, our data demonstrates that FtsHi5/TSL2 plays an important role in chloroplast development and protein accumulation in chloroplasts, especially at low environmental temperatures in Arabidopsis.

## Introduction

Chloroplast biogenesis affects plant growth and development due to its functions in photosynthesis and biosynthesis of amino acids, fatty acids, nucleotides, and phytohormones (Neuhaus and Emes, 2000). Chloroplasts in de-etiolated plants are developed from proplastids which are small and undifferentiated plastids. Upon light, the biogenesis of thylakoids begins immediately accompanied by the coordinated synthesis and assembly of proteins, chlorophylls and lipids in both space and time (Cackett et al., 2021). The plastid retains a functional genome which encodes about 100 proteins, while most of the other ∼2,000–3,000 proteins within chloroplasts are imported from the cytosol via the translocon at the outer envelope membrane of chloroplasts (TOC) and translocon at the inner envelope membrane of chloroplasts (TIC) systems (Li and Chiu, 2010; Jarvis and Lopez-Juez, 2013). Light-independent chloroplast development requires the expression of hundreds of chloroplast-related genes and photosynthesis associated nuclear genes. Protein homeostasis (proteostasis) in chloroplasts, maintained by a balance between protein import form cytosol, synthesis and degradation within chloroplasts, is essential for its functions during plant growth, development, and stress resistance (Sun et al., 2021).

Light-regulating chloroplast development requires transcription factors such as ELONGATED HYPOCOTYL 5 (HY5), GOLDEN2-LIKEs (GLKs), GATA NITRATE-INDUCIBLE CARBON-METABOLISM-INVOLVED (GNC) which appear to regulate the expression of genes essential for chloroplast development, chlorophyll biosynthesis, and photosynthesis (Chen et al., 2016; Gangappa and Botto, 2016; Cackett et al., 2021). At the same time, chloroplast development is regulated by environmental temperatures. In *Arabidopsis thaliana*, mutation of *DELAYED GREENING1* (*DG1*) impairs chloroplast RNA editing and chloroplast development at elevated temperature (29°C) (Sun et al., 2020). DG1 is a P-type PPR protein that interacts with multiple organellar RNA editing factor 2 (MORF2) and DYW1/2 involved in chloroplast RNA editing (Sun et al., 2020). Low temperature (less than 18°C) was also shown to inhibit the biogenesis and development of chloroplasts during the early seedling greening stage in rice (*Oryza sativa*) (Wang et al., 2016; He et al., 2020; Zhao et al., 2020). CP31A, a chloroplast ribonucleoprotein, is required for chloroplast development by maintaining mRNA stability and RNA editing under cold stress conditions (8°C) in Arabidopsis (Tillich et al., 2009; Kupsch et al., 2012). DG1 and CP31A are both involved in chloroplast RNA metabolism, while other types of factors connecting chloroplast development and temperatures are needed to be elucidated.

Filamentation temperature sensitive protein H (FtsH) protease belongs to a member of the ATPases associated with diverse cellular activities (AAA) protease family and is conserved among bacteria, mitochondria, and chloroplasts (Kato and Sakamoto, 2018). FtsH protease consists of one or more N-terminal transmembrane (TM) domain, a AAA domain and a C-terminal zinc-metalloproteinase domain. The thylakoid membrane-associated FtsH proteases FtsH2 and FtsH5, main parts of a complex as an ATP-dependent zinc metallopeptidase, function in the degradation of photodamaged photosystem II (PSII) reaction center proteins D1 and D2 (Kato et al., 2009; Dogra et al., 2019). Besides the FtsH protease, the Arabidopsis genome also has five FtsH-like (FtsHi1–FtsHi5) proteins without proteolytic activities due to lacking the zinc-binding motif (Wagner et al., 2012). Until recently, the functions of FtsHis have been revealed. Four FtsHi proteins (FtsHi1, FtsHi2, FtsHi4, and FtsHi5) as well as FtsH12 and Ycf2 form a large protein complex associating with TIC complex at the inner envelope membrane (IEM) to drive importing pre-proteins (Kikuchi et al., 2018). In this study, we characterized a chloroplast development defective mutant of Arabidopsis, *thermo-sensitive mutant in leaf color 2* (*tsl2*), which was obtained from an ethyl methane sulfonate (EMS) mutant screening. The *tsl2* mutant has chloroplast biogenesis defects at low ambient temperatures and *TSL2* is allelic to *FtsHi5*, encoding a FtsH-like protein lacking the zinc-binding motif. Further proteomic analyses revealed that chloroplast ultrastructure and protein homeostasis are impaired in *tsl2* mutant especially at low ambient temperatures. Thus, our results demonstrate the important role of TSL2/FtsHi5 in protein accumulation in chloroplast, which is associated with chloroplast development especially at low temperatures.

## Materials and Methods

### Plant Materials and Growth Conditions

All the *Arabidopsis thaliana* plants used in this study were in the Columbia (Col-0) background. The seeds were surface sterilized for 5 min with 75% ethanol and then were washed three times with sterilized water. After sterilization, seeds were sown on 1/2 Murashige and Skoog (MS) medium (containing 1.2% sucrose and 0.8% agar, pH 5.7) and stratified at 4°C for 2 days. Then they were transferred to 22°C under a 16-h light/8-h dark cycle. For phenotypic assays, seedlings were firstly grown at 22°C, and then kept at 22°C or transferred to different temperature conditions as indicated.

### Measurement of Chlorophyll Content

Fresh leaves of plants were weighted and immediately incubated in 1 mL of 95% ethanol in 1.5 mL microfuge tubes at 4°C in the dark. After 24 h, the absorbance of the supernatant was measured at 649 and 665 nm. The chlorophyll concentration (CT) was the sum of chlorophyll a (13.95 × OD_665_−6.88 × OD_649_) plus chlorophyll b (24.96 × OD_649_−7.32 × OD_665_). Content of chlorophyll = (CT × V)/W, where V is the volume of 95% ethanol and W is the weight of fresh tissues. Each sample performed with three independent replicates.

### Bulk Segregant Analysis

For genome resequencing, the *tsl2* mutant in Col-0 background was crossed to the WT Landsberg (Ler) ecotype. About three hundred seedlings whose leaves were yellow at 16°C from the F2 segregation population were sampled, and their genomic DNA was pooled for deep sequencing using the Illumina HiSeq 4000 platform (Majorbio, China) according to the standard Illumina protocol.

### Plasmid Construction

For the complementation of *tsl2* mutants, the 8.5-kb wild-type genomic DNA fragment of AT3G04340 including the upstream promoter and coding sequences was amplified by PCR and inserted into the pCAMBIA1300 plasmid. The construct was subsequently transformed into *Agrobacterium tumefaciens* strain GV3101 via the freeze-thaw method and introduced into *tsl2* mutant plants via the floral dip method. All the primers used in the present study are listed in Supplementary Table 1. For the expression of *TSL2-FLAG*, the promoter and the coding sequence of *TSL2* were amplified and inserted into pCAMBIA1306, respectively, and the FLAG tag was fused to TSL2 at the C-terminus. The mutated form of *TSL2m* was amplified from cDNA derived from *tsl2* mutant plants. Both plasmids were transformed into GV3101 and introduced into WT plants via the floral-dip method. Primers were listed in Supplementary Table 1.

### Transmission Electron Microscopy

For analysis of the chloroplast ultrastructure, WT and *tsl2* mutant seedlings were grown at 22°C for 4 days and then transferred to 16 and 29°C or maintained at 22°C for 10 days. Subsequently, true leaves were detached and fixed overnight at 4°C in 2.5% glutaraldehyde and 0.1 M phosphate buffer (pH 7.0) and processed for electron microscopy as described (Hyman and Jarvis, 2011). Ultra-thin sections were cut with an ultra-microtome (LEICA EM UC7) and observed under a transmission electron microscope (Hitachi H-7650).

### Gene Expression Analysis

Total RNA was extracted from various genotypes using an RNA Prep Pure Plant kit (Tiangen, China). For reverse transcription, 2 μg of total RNA with oligo(dT) and random 6-mer primers were used to synthesize cDNA in a 20 μL reaction using M-MLV reverse transcriptase (TaKaRa, China). The cDNA was used to perform RT-PCR and qRT-PCR. qRT-PCR was performed using the SuperReal PreMix Color kit (Tiangen, China) in a CFX96 real-time system (Bio-Rad, United States). Each comparison consisted of three biological replicates, and the expression of *PP2A* served as an internal control. For RT-PCR, the expression of *UBQ5* was used as an internal control.

### Protein Extraction and Immunoblotting Analysis

Total proteins were extracted from plants with extraction buffer [125 mM Tris–HCl (pH 8.0), 375 mM NaCl, 2.5 mM EDTA, 1% SDS, and 1% β-mercaptoethanol], separated by 10% SDS-PAGE gels, and analyzed using anti-FLAG (Abmart, China) or anti-Tubulin (Abmart, China).

### Intact Chloroplast Isolation

Chloroplasts were isolated according to previous methods (Rensink et al., 1998; Macko et al., 2002) with modifications. Two-week-old (26°C) and 3-week-old (18°C) Arabidopsis plants were homogenized in ice-cold grinding buffer (330 mM sorbitol, 30 mM Tricine-KOH, pH 8.4, 5 mM EGTA, 5 mM EDTA, 10 mM NaHCO_3_). The suspension was filtered through three layers of Miracloth (Calbiochem, United States) and centrifuged for 5 min at 1,500 *g* to pellet the crude chloroplast fraction. The pellet was resuspended in 300 mM sorbitol, 20 mM HEPES-KOH (pH 7.6), 5 mM MgCl_2_, and 2.5 mM EDTA. The suspension was layered on top of a step gradient consisting of (bottom to top): 2 mL of 80% (v/v) Percoll/grinding buffer, 4 mL of 35% (v/v) Percoll/grinding buffer in a 15-mL polycarbonate tube. The gradient was centrifuged for 25 min at 3,000 *g* and then the lower band was collected and diluted with 3 volumes of dilution buffer (330 mM sorbitol, 50 mM HEPES-KOH, pH 8.0, 2 mM DTT). The chloroplasts were centrifuged for 2 min at 1,000 *g*. The pellet was resuspended in 25 mL dilution buffer and centrifuged for 1 min at 1,000 *g*. The chloroplast pellet was resuspended in 300 μL Lysis buffer (8 M urea, 1% Triton X-100, 10 mM dithiothreitol, 1% protease inhibitor cocktail, and 1% phosphatase inhibitor, 2 mM EDTA).

### Protein Extraction, Digestion, and Tandem Mass Tag Labeling

Proteomics analyses were carried out in the company PTM BIO (Hangzhou, China). Briefly, the chloroplast pellet was resuspended in 300 μL Lysis buffer (8 M urea, 1% Triton X-100, 10 mM dithiothreitol, 1% protease inhibitor cocktail, and 1% phosphatase inhibitor, 2 mM EDTA), followed by sonication three times on ice using a high intensity ultrasonic processor (Scientz, China) and centrifugation at 20,000 *g* at 4°C for 10 min. The supernatant was precipitated with cold 20% trichloroacetic acid for 2 h at −20°C. After centrifugation at 12,000 *g* at 4°C for 10 min, the supernatant was discarded and the remaining precipitate was washed with cold acetone for three times. Finally, the protein was dissolved in 8 M urea and the protein concentration was determined with a BCA kit, according to the manufacturer’s instructions. Before digestion, the protein sample was diluted by adding 100 mM triethylammonium bicarbonate (TEAB). For digestion, trypsin was added at 1:50 trypsin-to-protein mass ratio for digestion overnight and the protein solution was reduced with 5 mM DTT for 30 min at 56°C and then alkylated with 11 mM iodoacetamide for 15 min at room temperature in the dark. For TMT labeling, peptide was desalted with Strata X C18 SPE column (Phenomenex, United States) and vacuum dried. After that, peptide was reconstituted in 0.5 M TEAB and processed according to the manufacturer’s protocol for the TMT kit. Briefly, one unit of TMT reagent was thawed and reconstituted in acetonitrile and added to the peptide sample. Then, the peptide mixtures were incubated for 2 h at room temperature and pooled, desalted, and dried by vacuum centrifugation.

### High Performance Liquid Chromatography Fractionation and Liquid Chromatography Tandem Mass Spectrometry Analysis

High performance liquid chromatography (HPLC) fractionation was performed with high pH reverse-phase HPLC. Briefly, peptides were dissolved in 0.1% formic acid, and then directly loaded onto reversed-phase analytical columns (Agilent 300Extend C18). The peptides have a stepwise gradient of 8∼32% acetonitrile, pH 9.0, 60 min to separate 60 components, then the peptides were combined into six components, and vacuum freeze-dried for subsequent analyses. The peptides are dissolved in the mobile phase A (0.1% formic acid and 2% acetonitrile) of liquid chromatography and then separated using the EASY-nLC 1200 ultra-high-performance liquid system. The gradient was increased from 7 to 11% (0.1% formic acid in 90% acetonitrile) over 4 min, 11 to 32% in 49 min, 32 to 80% in 4 min, and then held at 80% for the last 3 min, all at a constant flow rate (500 nL/min) on an EASY-nLC 1000 UPLC system. The peptides were sent to NSI source followed by tandem mass spectrometry (MS/MS) in Orbitrap Exploris 480 (Thermo Fisher Scientific, CA, United States) coupled online to the UPLC in the Orbitrap with routine setting parameters. The resulting MS/MS data were processed using the Proteome Discoverer (v2.4.1.15) against the Arabidopsis TAIR database^1^. The mass error for precursor ions was less than 20 ppm in the first search and 5 ppm in the main search, and the mass error for fragment ions was less than 0.02 Da. Differentially regulated peptides were calculated and their corresponding proteins were subjected to GO analysis (Biological Process, Cellular Component, Molecular Function).

## Results

### The *tsl2* Mutant Has a Temperature-Sensitive Phenotype

**FIGURE 1:**
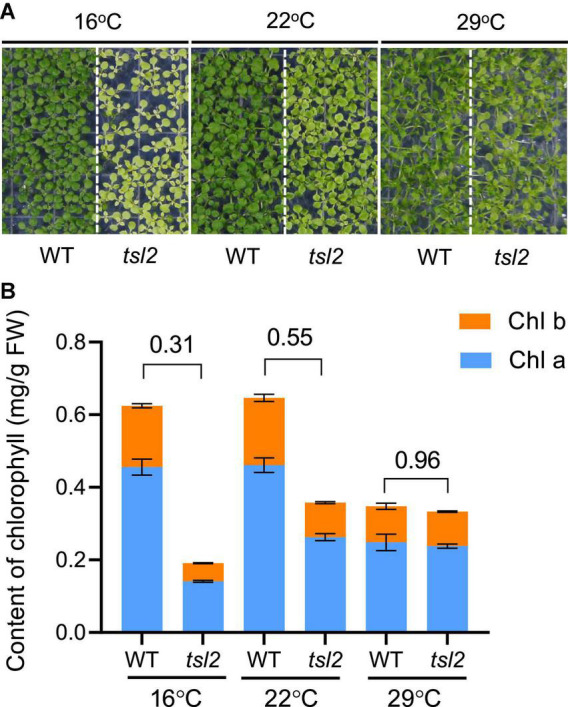
The chloroplast development of *tsl2* mutant is sensitive to cold temperatures. **(A)** Temperature-sensitive phenotypes of the *tsl2* mutant plants. Wild-type (WT) and *tsl2* mutant seedlings grown under normal growth temperature (22°C) for 4 days were kept at 22°C or transferred to 16 or 29°C for 10 days and then photographed. **(B)** Analysis of chlorophyll contents of plant leaves grown under different temperature conditions. The data represents the average of samples and the error bars indicate the standard error (*n* = 3).

To study the effects of temperature on plant growth and chloroplast development, we identified a mutant *thermo-sensitive mutant in leaf color 2* (*tsl2*) through screening an EMS-mutated population of *Arabidopsis* plants. The *tsl2* mutant showed a weak yellowish phenotype at 22°C (Figure 1A). The phenotype was more pronounced when the growth temperature was reduced to 16°C, and the leaf color of *tsl2* was similar to that of the wild type (WT) when the temperature was increased to 29°C (Figure 1A). Thus, *tsl2* mutant has a temperature-sensitive phenotype.

To further characterize the *tsl2* mutant, we examined the chlorophyll contents in *tsl2* and WT seedlings grown at different temperature conditions. Consistent with the leaf color defects, the content of chlorophyll of *tsl2* was reduced to 31% of that in the WT at 16°C (Figure 1B). As the temperature increased to 22°C, the difference between *tsl2* and WT decreased to 55%. At 29°C, the chlorophyll content in *tsl2* was similar to that of WT (Figure 1B). These results demonstrated that the lower chlorophyll content and yellowish phenotype of the *tsl2* mutant is enhanced under low temperature conditions.

### *TSL2* Encodes the FtsH-Like/FtsH-Inactive Protein FtsHi5

**FIGURE 2:**
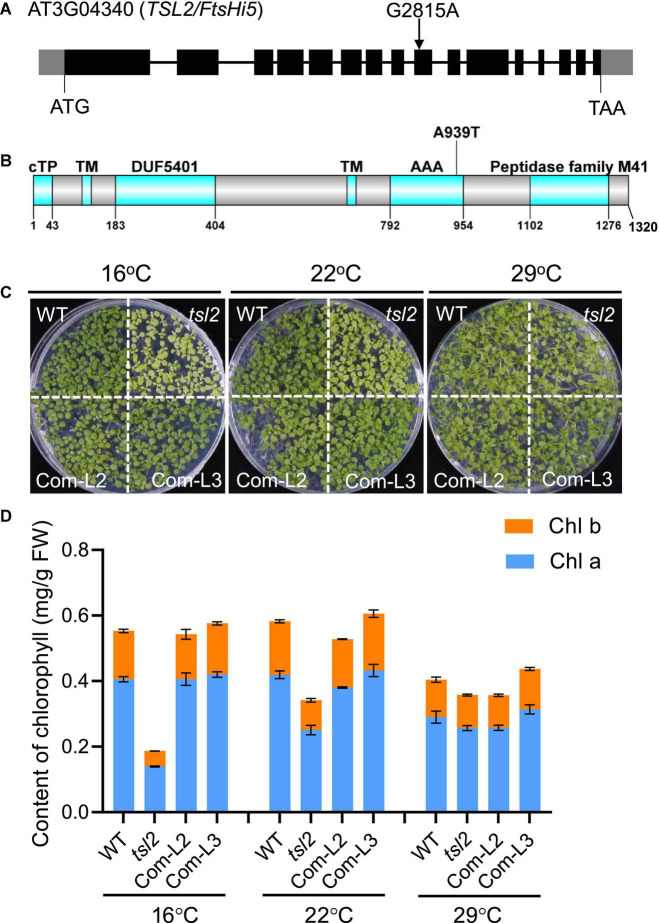
The *tsl2* mutant phenotype is complemented by *TSL2*/*FtsHi5*. **(A)** Gene structure and mutation sites of *TSL2*/*FtsHi5* in the *tsl2* mutant. Gray boxes represent the 5′- and 3′-untranslated regions, black boxes represent exons and lines represent introns. **(B)** Protein domains of TSL2. cTP, chloroplast transit peptide; TM, transmembrane domain; DUF5401, the domain of unknown function 5401; AAA, ATPase family associated with various cellular activities. **(C,D)** Phenotypic analysis of the complemented lines (Com-L2 and Com-L3). The data represents the average of samples and the error bars indicate the standard error (*n* = 3).

To identify the mutated gene responsible for the *tsl2* phenotype, we crossed *tsl2* to Ler to generate an F2 population. The segregation ratio of the green and yellowish seedlings in the F2 population was about 3:1, suggesting that the abnormal phenotypes of the *tsl2* resulted from mutation of a single nuclear gene. We selected plants with leaf developmental defects at 16°C in the F2 segregation population for whole-genome resequencing. Bulked segregant analysis (BSA) showed that there was a guanine (G) to an adenine (A) transition at the 9th exon of *FtsHi5* (AT3G04340) in the *tsl2* mutant, which led to an amino acid change from Ala to Thr (A939T) in AAA domain of the protein (Figures 2A,B).

To test whether the mutation of AT3G04340 is responsible for the *tsl2* mutant phenotype, we performed a transgenic complementation experiment. The 8.5-kb WT genomic sequence of AT3G04340, including its promoter and coding region, was transferred into the *tsl2* mutant. Phenotypic analysis demonstrated that AT3G04340 complements the temperature-sensitive phenotype and the chlorophyll content defects observed in *tsl2* mutant plants (Figures 2C,D). Thus, AT3G04340 corresponds to *TSL2* which encodes FtsHi5, a proteolytically inactive FtsH member in Arabidopsis. Homozygous mutants of *ftshi5* had been previously found to be seed-lethal (Meinke, 2020). Therefore, *tsl2* identified in this study is a temperature-conditioned week allele of *ftshi5*.

### TSL2 Is Responsible for Chloroplast Development at Low Temperature

**FIGURE 3:**
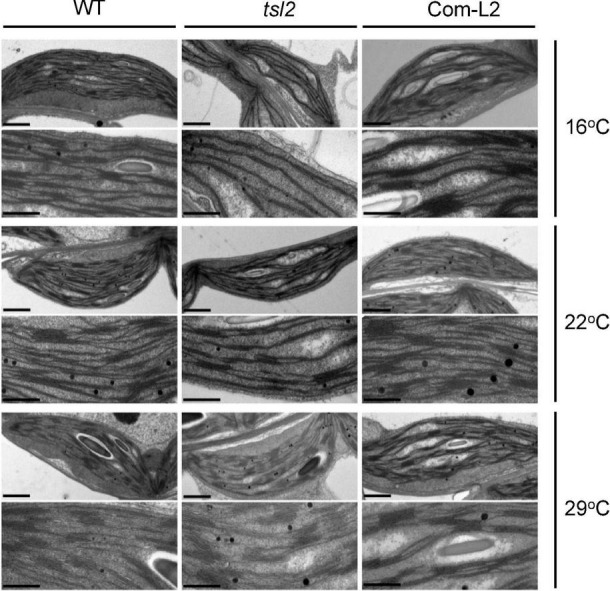
The temperature-associated chloroplast developmental defects of *tsl2* mutant are rescued by *TSL2*/*FtsHi5*. Wild-type (WT), *tsl2* mutant, and genetically complemented transgenic plant (Com-L2) grown under normal growth temperature (22°C) for 4 days were kept at 22°C or transferred to 16 or 29°C for 10 days, and then the ultrastructure of chloroplasts was examined under transmission electron microscopy. Scale bars, 1 μm (upper), 0.5 μm (lower).

To examine the detailed chloroplast developmental defects, we observed chloroplast ultrastructure in mesophyll cells of seedlings grown at different temperatures between WT and *tsl2* mutant plants by transmission electron microscopy (TEM). Under normal growth temperature condition (22°C), chloroplasts in the *tsl2* mutant showed less organized thylakoid membrane systems compared to that of the WT plants (Figure 3). At 16°C, there were few mature chloroplasts in the *tsl2* mutant, whereas chloroplasts with dense and well-structured grana lamella stacks were observed in WT plants. As the temperature increased to 29°C, the chloroplast of *tsl2* showed similar thylakoid membrane structures to that of the WT plants (Figure 3). The temperature-associated chloroplast developmental defects of *tsl2* mutant were rescued by *TSL2*/*FtsHi5* (Figure 3). These results demonstrated that the reduced chlorophyll content and yellowish phenotype of the *tsl2* mutant observed under low temperature conditions is resulted from abnormal chloroplast development.

### *TSL2* Mutation in *tsl2* Mutant Doesn’t Affect the Accumulation of Either *TSL2* Transcript or TSL2 Protein

**FIGURE 4:**
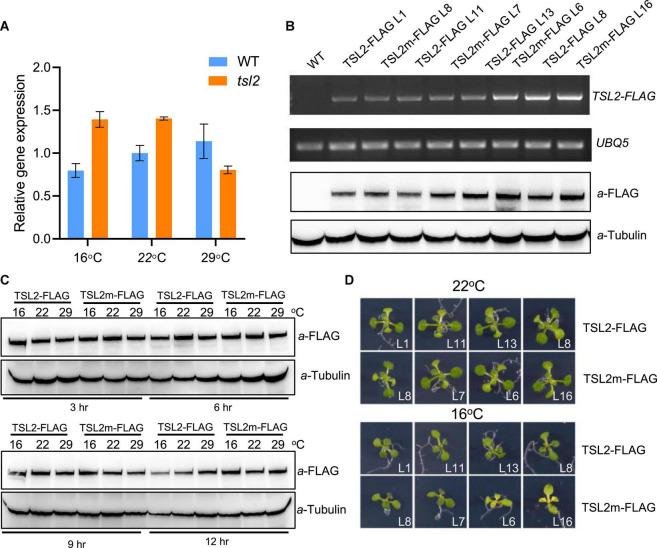
Mutation of *TSL2* does not affect TSL2 protein accumulation. **(A)** RT-qPCR detection of *TSL2* expression in wild-type (WT) and *tsl2* mutant plants. The seedlings grown at 22°C for 4 days were kept at 22°C or transferred to 16 or 29°C for 7 days, and collected for RT-qPCR analysis. The *TSL2* expression was normalized to the WT sample (22°C), both of which were normalized to the expression of *PP2A*. Error bars represent SE (*n* = 3). **(B,C)** Protein accumulation of TSL2-FLAG and TSL2m-FLAG. Various transgenic lines grown at 22°C for 10 days were collected for RT-PCR and western blotting analysis. *UBQ5* gene was used as the control. Transgenic plants (TSL2-L1 and TSL2m-L8) grown at 22°C for 10 days were transferred to 16, 22, or 29°C for the indicated time, and total proteins were extracted for western blotting. Tubulin was used as a loading control. **(D)** Phenotype of *TSL2-FLAG* and *TSL2m-FLAG* overexpression lines. The transgenic plants were grown at 22°C for 4 days and transferred to 16°C or kept in 22°C for 10 days and photographed. The mutated form of *TSL2* (A939T) was amplified from the *tsl2* mutant.

The chloroplast development defects of *tsl2* mutant is temperature-sensitive, which prompted us to investigate whether low temperature affects *TSL2* transcript accumulation in *tsl2* mutant. So, we performed quantitative real-time polymerase chain reaction (qRT-PCR) analysis to detect the expression of *TSL2* in WT and *tsl2* plants grown under different temperature conditions. The results showed that there was no obvious reduction in terms of *TSL2* transcript level in *tsl2* to that of WT at 16 or 22°C, at which temperature the *tsl2* showed defects in chloroplast development (Figure 4A). Next, we checked the protein accumulation level of the WT (TSL2) and mutated form (TSL2m) of TSL2 by expressing the FLAG-tagged proteins driven by the native *TSL2* promoter in the WT background, respectively. We selected several lines with similar expressing levels of *TSL2* as detected by RT-PCR analysis in each form (Figure 4B), and then detected the TSL2 protein levels by Western blotting analysis. The results demonstrated that mutation of TSL2 (A939T) does not affect the protein accumulation of TSL2 at 22°C (Figure 4B). Furthermore, Mutation of *TSL2* did not affected its protein level at 16 or 29°C (Figure 4C). Interestingly, the TSL2m lines with higher expression levels had yellowish leaves at 16°C but normal leaves at 22°C (Figure 4D), demonstrating that high accumulation of TSL2m represses chloroplast development in a temperature-dependent manner. Taken together, mutation of TSL2 does not affect the protein accumulation of TSL2/FtsHi5, but possibly the protein activity of TSL2/FtsHi5 at low temperatures.

### Chloroplast Protein Accumulation Is Affected in *tsl2* Mutant Under Low Temperature Conditions

**FIGURE 5:**
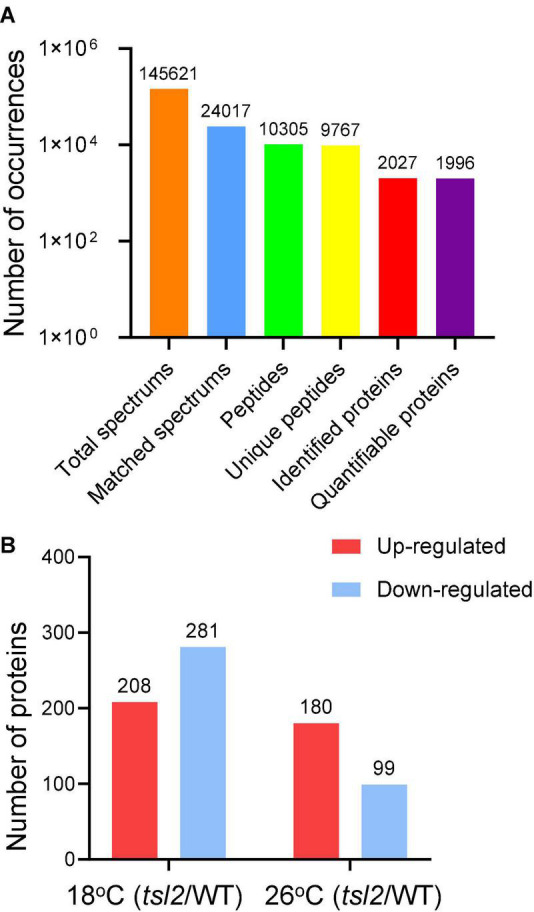
Quantitative proteomic study of the effect of *TSL2* mutation on chloroplast proteome. **(A,B)** Basic information on the proteomic analysis. The wild-type (WT) and *tsl2* mutant plants grown under normal growth temperature (22°C) for 4 days were transferred to 18°C or 26°C for 12 days and harvested for chloroplast isolation and further proteomic analysis. Basic information on proteomics was shown in **(A,B)**.

**FIGURE 6:**
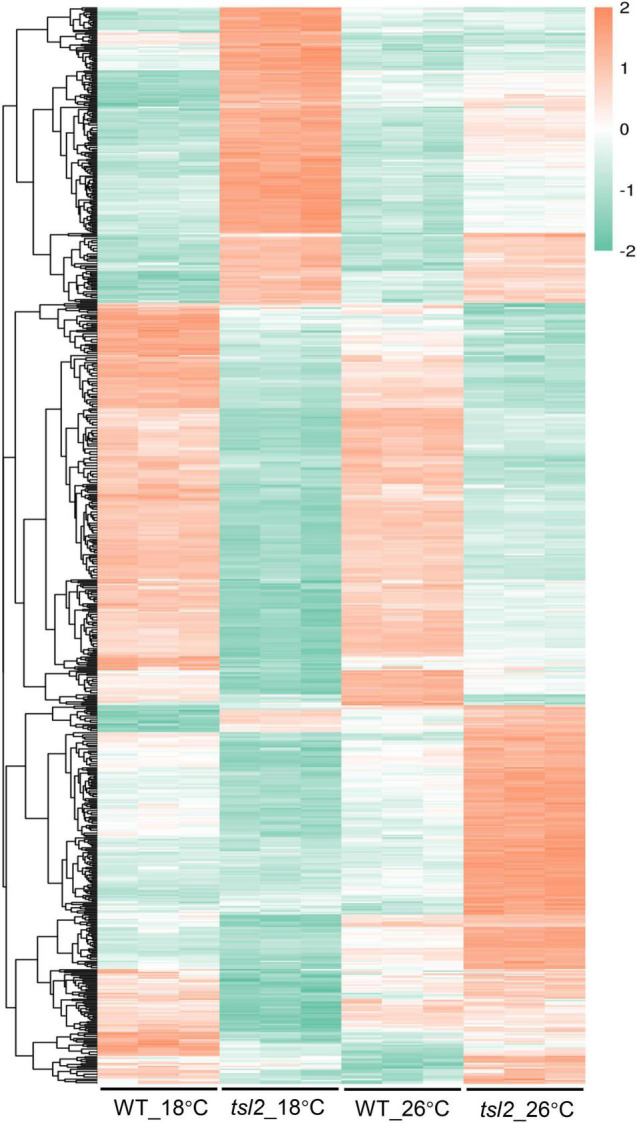
Quantitative proteomic study identifies differentially expressed proteins between WT and *tsl2* mutant plants. Each horizontal bar represents the level of an identified protein. Only those proteins with 1.3 ≤ FC or FC ≤ 0.7 (*P* ≤ 0.05) were used for heat map visualization.

FtsHi5 and other FtsHis were shown to form Ycf2-FtsHi heteromeric AAA-ATPase complex which is required for chloroplast protein import in Arabidopsis (Kikuchi et al., 2018). To assess chloroplast proteome of *tsl2* mutant at low and high temperature, we employed TMT-based quantitative proteomics to compare the chloroplast proteins in WT and *tsl2* plants after chloroplast protein isolation. To obtain enough true leaves and avoid high temperature stress, we chose 18 and 26°C as low temperature and high temperature growth conditions, respectively. The *tsl2* mutant still showed chloroplast developmental defects at 18°C and largely recovered at 26°C (Supplementary Figures 1A,B). In total, 10,305 peptides, corresponding to 2,027 proteins were identified in chloroplast fractions from WT and *tsl2* plants at 18 and 26°C, among which 1,996 proteins contained quantifiable information (Figure 5A and Supplementary Table 2). Principal components analysis (PCA) results showed a good reproducibility of data for the samples in each group (Supplementary Figure 2). The subcellular localization prediction based on the normalized protein abundance scale (NPAS) available in the SUBA4 toolbox (Hooper et al., 2017) found that 79.62% proteins are localized in plastids/chloroplasts, 14.46% proteins are unassigned, and 6.22% proteins are localized in other compartments (Supplementary Figure 3), consistent with the samples originated from isolated chloroplasts. Volcano plot and protein abundance data showed the differential expression of proteins between WT and *tsl2* at 18 and 26°C (Supplementary Figure 4 and Supplementary Table 3). Among all the quantifiable proteins, there were 208 proteins that were upregulated in *tsl2* compared with that in WT (Fold change >1.3, *p* < 0.05) and 281 proteins that were downregulated in *tsl2* at 18°C (Fold change <0.7, *p* < 0.05). While at 26°C, there were 180 proteins upregulated and 99 proteins downregulated in *tsl2* mutant, respectively (Figures 5B, 6 and Supplementary Table 3). These results suggested that TSL2 is required for chloroplast protein homeostasis under low temperature conditions.

**FIGURE 7:**
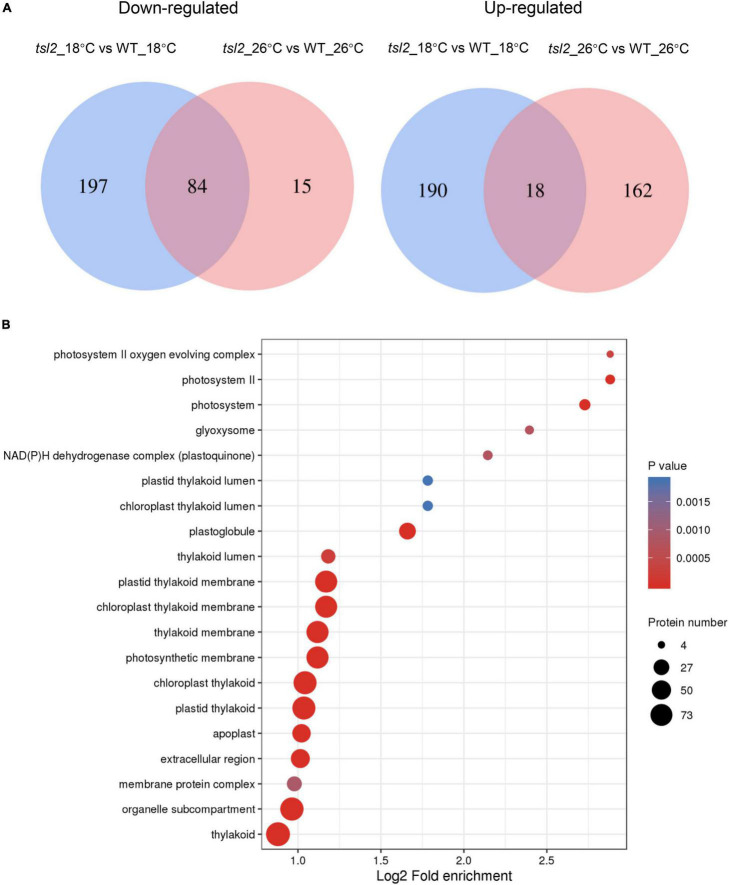
TSL2 regulates chloroplast protein accumulations. **(A,B)** Proteomic analysis of chloroplast proteins in WT and *tsl2* mutant plants. Venn diagrams show the numbers of overlapping and non-overlapping proteins that were upregulated or downregulated under two temperature conditions **(A)**. Criteria for differential expression were set as *q* ≤ 0.05, fold change (FC) ≥1.3 for upregulation or FC ≤ 0.7 for downregulation. GO analysis of 281 downregulated proteins in *tsl2* mutant at 18°C is shown in **(B)**.

In order to understand the observed differential chloroplast protein of *tsl2* between 18 and 26°C, we compared the differentially upregulated or downregulated proteins. Venn diagrams showed that there were 197 proteins downregulated only at 18°C, while the 15 proteins were specifically downregulated at 26°C (Figure 7A and Supplementary Data Sheet 1). There were 18 proteins that were commonly upregulated, and 190 proteins and 162 proteins were specifically upregulated at 18 and 26°C, respectively (Figure 7A and Supplementary Data Sheet 1). We considered these 281 proteins that were downregulated in *tsl2* mutant at 18°C were *TSL2*-dependent proteins associated with the observed temperature-sensitive phenotype. These proteins were enriched in GO terms associated with chloroplast/plastid thylakoid membrane, chloroplast/plastid thylakoid, photosynthetic membrane, and photosystem (Figure 7B), which was consistent with their role in chloroplast development and function. Those 208 upregulated proteins in *tsl2* mutant at 18°C were enriched in GO terms associated with chloroplast/plastid stroma and ribosomes (Supplementary Figure 5). Among the *TSL2*-dependent proteins, the chlorophyll a oxygenase (CAO, AT1G44446) involved in chlorophyll b biosynthesis (Espineda et al., 1999) and CURVATURE THYLAKOID 1C (CURT1C), a member of CURT1 protein family which promotes organized thylakoid biogenesis in Arabidopsis (Armbruster et al., 2013; Sandoval-Ibanez et al., 2021), was reduced in *tsl2* mutant plants at 18°C but not at 26°C (Supplementary Data Sheet 1). Other factors involved in chloroplast development and function were also found to be *TSL2*-dependent, including components of the light harvesting complex (LHCA5 and LHCB1), NAD(P)H-quinone oxidoreductase subunits (ndhS and ndhU), Photosynthetic NAD(P)H dehydrogenase subunits (NDH18 and NDH48), Photosystem I reaction center subunits (psaD, psaE, psaF, and psaG) (Supplementary Data Sheet 1). Therefore, TSL2 is required for chloroplast development by maintaining protein accumulation in chloroplasts related to photosynthesis and chloroplast biogenesis.

## Discussion

Plant FtsH proteins are evolved from the cyanobacterial membrane-bound AAA-protease FtsH that plays roles in the quality control of integral membrane proteins (Dalbey et al., 2012; Langklotz et al., 2012). In Arabidopsis, the chloroplast proteomes also contain five FtsHi (FtsH-inactive) proteins lacking the zinc binding motif (His-Glu-X-X-His) which is essential for proteolytic activity (Wagner et al., 2012; Mishra and Funk, 2021). A heteromeric complex comprised of FtsH12, FtsHi1, FtsHi2, FtsHi4, FtsHi5, pdNAD-MDH, and Ycf2 was found as an import motor for protein import at IEM (Kikuchi et al., 2018). In this study, we identified a point mutation of *FtsHi5* and carried out quantitative proteomic analysis of *tsl2* mutant, further demonstrated the important function of FtsHi5/TSL2 in chloroplast protein homeostasis. Our current results further revealed substantial changes at chloroplast proteome level in *tsl2* mutant plants at 18°C, which is associated with chloroplast development and function. Similar pale-green phenotypes with defects in chloroplast have also been found in knock-down lines of *FtsHi4* (Lu et al., 2014), *FtsH12* (Mielke et al., 2021) and *pdNAD-MDH* (Schreier et al., 2018), and in *ftshi1* carrying a point mutation in *FtsHi1* (Kadirjan-Kalbach et al., 2012), which is consistent with the association of FtsHi5/TSL2 with the protein complex in chloroplast development.

Like all other ATP-dependent proteases, the function ATPase domain is essential for substrate recognition, unfolding and translocation to the proteolytic sites (Jessop et al., 2021). The ATPase domain can confer chaperone activity that is independent of the proteolytic activity. Mutation of FtsHi5 (A939T) in *tsl2* is located in the AAA-ATPase domain. Expression of *TSL2m* in the WT resulted in yellowish true leaves, indicating the point mutation in *tsl2* mutant impairs substrate/client protein recognition, and the mutated protein may interfere with the formation and/or function of the protein complex. It would be interesting to determine the stoichiometry or subunit arrangement of the FtsH12-FtsHi-pdNAD-MDH motor complex. FtsHi5/TSL2 also contains the domain of unknown function 5401 (DUF5401). The previously reported *ftshi5-1* mutant containing a mutation (D400N) located near the DUF5401 domain also showed chloroplast development defects (Wang et al., 2018), indicating that DUF5401 is important for the function FtsHi5, while its function is not known. Among the five FtsHis, FtsHi5 is the only one that has a DUF5401 domain, revealing its unique feature in the complex. Previous study showed that the abundance of FtsH12 regulates the accumulation of the FtsHi subunits, but not pdNAD-MDH (Mielke et al., 2021). While in our current study, none of the proteins from the FtsH12-FtsHi-Ycf2 complex were downregulated in *tsl2* mutant at 18 or 26°C, suggesting FtsHi5/TSL2 is not involved in the accumulation of the FtsH12-FtsHi-Ycf2 complex in Arabidopsis.

FtsH protease was firstly discovered by screening temperature sensitive mutants in filamentation induction in yeasts, while the function of FtsH family proteins in temperature response in plants is still less understood. The thylakoid membrane-associated FtsH proteases FtsH2 (VAR2) and FtsH5 function in the degradation of photodamaged photosystem II (PSII) reaction center proteins D1 and D2 (Kato et al., 2009; Dogra et al., 2019). The *var2* mutant displays a chlorotic phenotype at 8°C compared to a leaf variegation phenotype at 22°C indicating that FtsH2 mediates chloroplast biogenesis in a temperature-dependent manner (Liu et al., 2010). The chloroplast envelope-integrated FtsH11 plays critical roles in maintaining the chloroplast development and thermotolerance to elevated temperatures (Chen et al., 2006, 2018; Wagner et al., 2016). The proteolytic activity of FtsH11 is essential for conferring thermotolerance in Arabidopsis and several potential substrates of FtsH11 were identified including CPN60 and Tic40 (Adam et al., 2019). These results suggest that distinct FtsH proteins are involved in responses to different ranges of temperatures. In the present study, FtsHi5/TSL2 has been shown to be required for chloroplast development at low temperatures. How FtsHi5/TSL2 mediates temperature responses? The expression of mutated *TSL2* and the protein accumulation of mutated TSL2 were not changed at low temperature. It is possible that mutation of FtsHi5/TSL2 affects the efficiency of importing of chloroplast proteins via the FtsH12-FtsHi-pdNAD-MDH protein complex at low temperatures. Another possibility is that the function of the FtsH12-FtsHi-pdNAD-MDH protein complex is reduced at low temperatures, and therefore the effect of FtsHi5/TSL2 mutation becomes more evident at low temperatures.

## Conclusion

Our results demonstrated the important function of FtsHi5/TSL2 in chloroplast protein accumulation, which is required for chloroplast development, especially at low environmental temperatures.

## Data Availability Statement

The mass spectrometry proteomics data have been deposited to the ProteomeXchange Consortium via the PRIDE partner repository with the dataset identifier PXD030185.

## Author Contributions

J-YL and J-XL designed the experiments, analyzed the data, and wrote the manuscript. J-YL, J-LS, and Y-YT performed the experiments. All authors contributed to the article and approved the submitted version.

## Conflict of Interest

The authors declare that the research was conducted in the absence of any commercial or financial relationships that could be construed as a potential conflict of interest.

## Publisher’s Note

All claims expressed in this article are solely those of the authors and do not necessarily represent those of their affiliated organizations, or those of the publisher, the editors and the reviewers. Any product that may be evaluated in this article, or claim that may be made by its manufacturer, is not guaranteed or endorsed by the publisher.

## Footnotes

1
https://www.arabidopsis.org/index.jsp

## Supplementary Material

Supplementary Table 1 List of primers used in this study.

Supplementary Table 2 MS/MS spectrum database search analysis summary.

Supplementary Table 3 Summary of number of differentially expressed proteins for each comparison.

Supplementary Data Sheet 1 Differentially expressed chloroplast proteins between WT and *tsl2* mutant plants.
