# Supplementary material for: The FtsH-Inactive Protein FtsHi5 Is Required for Chloroplast Development and Protein Accumulation in Chloroplasts at Low Ambient Temperature in Arabidopsis
Source: Front Plant Sci. 2022 Feb 3;12:830390. doi: 10.3389/fpls.2021.830390 (PMC8850778; doi:10.3389/fpls.2021.830390)
Supplement: Supplementary Data Sheet 1 — Differentially expressed chloroplast proteins between WT and tsl2 mutant plants. [file Data_Sheet_1.pdf]

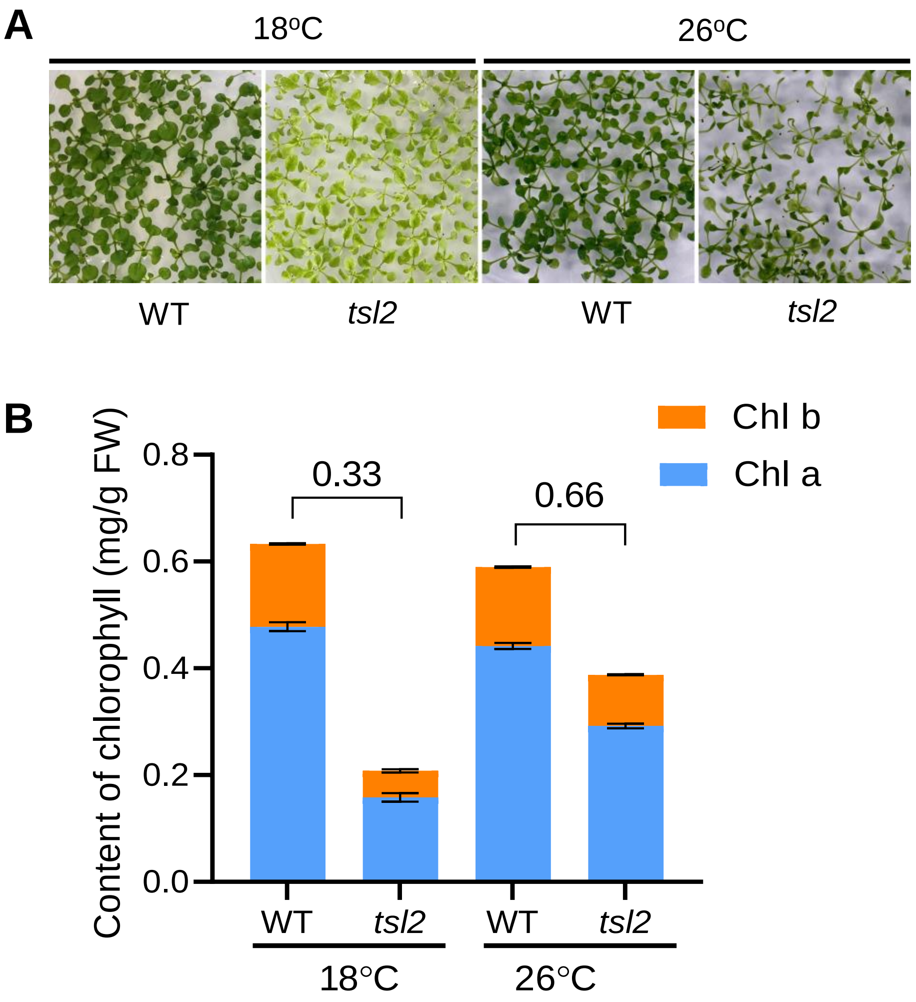


**Fig. S1. Phenotype of the *tsl2* mutant at 18ºC and 26ºC.**
(**A-B**) Phenotypes of the mutant plants. The wild-type (WT) and *tsl2* mutant grown under normal growth temperature (22ºC) conditions for 4 days were transferred to 18ºC or 26ºC for 12 days and photographed (A), chlorophyll contents of plant leaves were measured (B). The data represents the average of samples and the error bars indicate the standard error (n = 3).

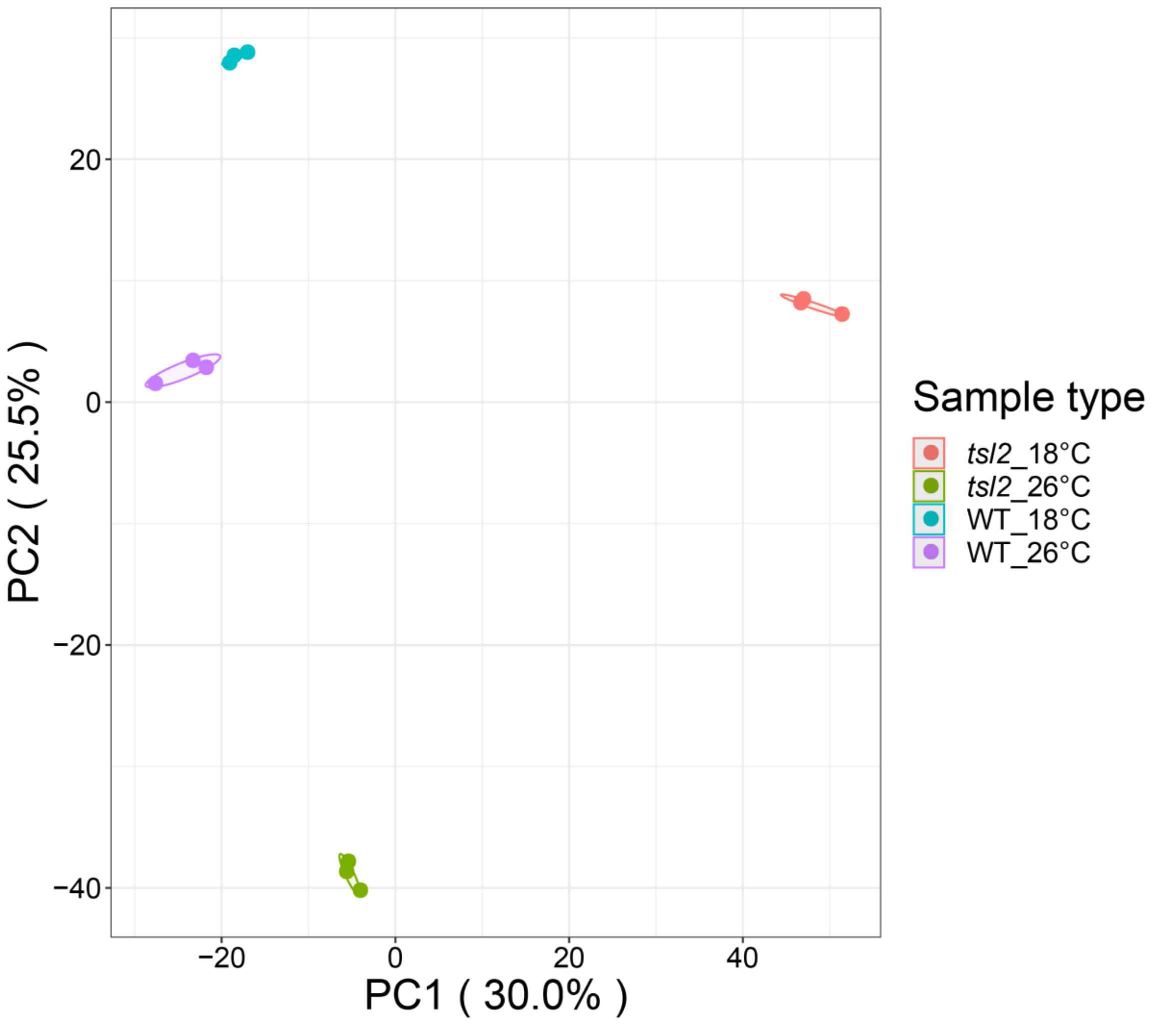


**Fig. S2. Principle Component Analysis (PCA) of samples for quantitative proteomics.**

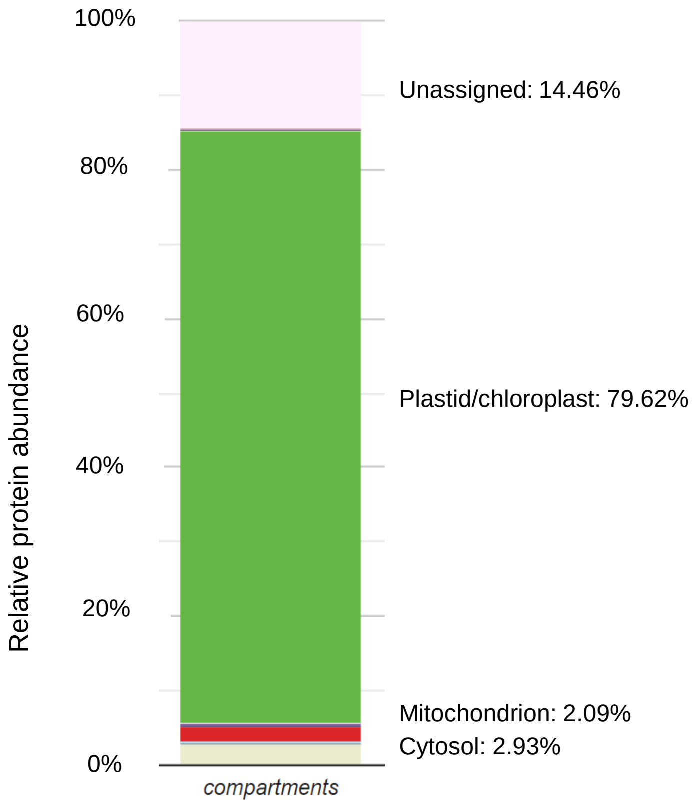


**Fig. S3. Gene Ontology (GO) analysis of subcellular localizations of quantifiable proteins identified in the proteomics study.**

Protein subcellular localizations were predicted by SUBA4 using the multiple marker abundance profiling (MMAP) online tool (https://suba.live/toolbox-app.html).

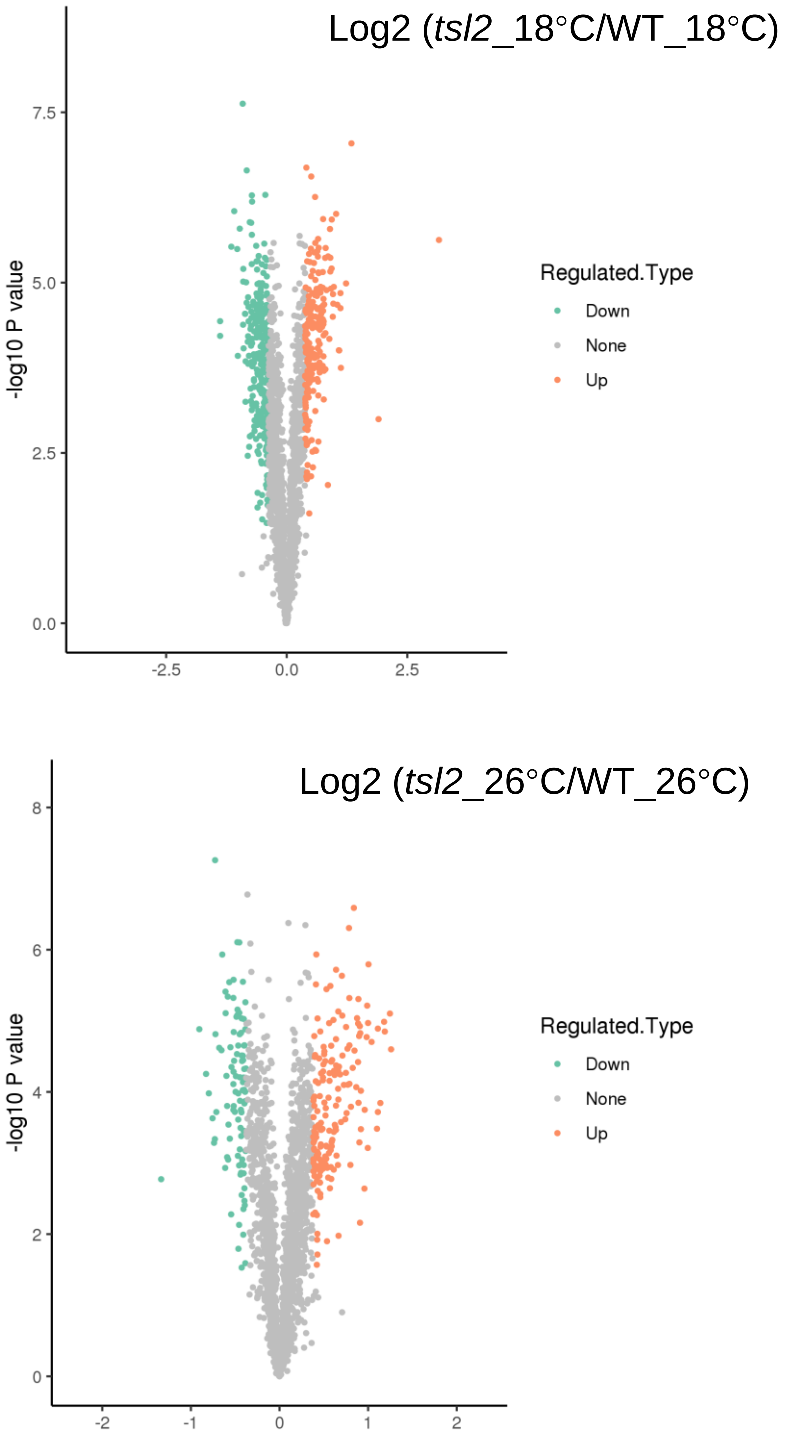


**Fig. S4. Differentially expression proteins between WT and *tsl2* mutant plants under two temperature conditions.**

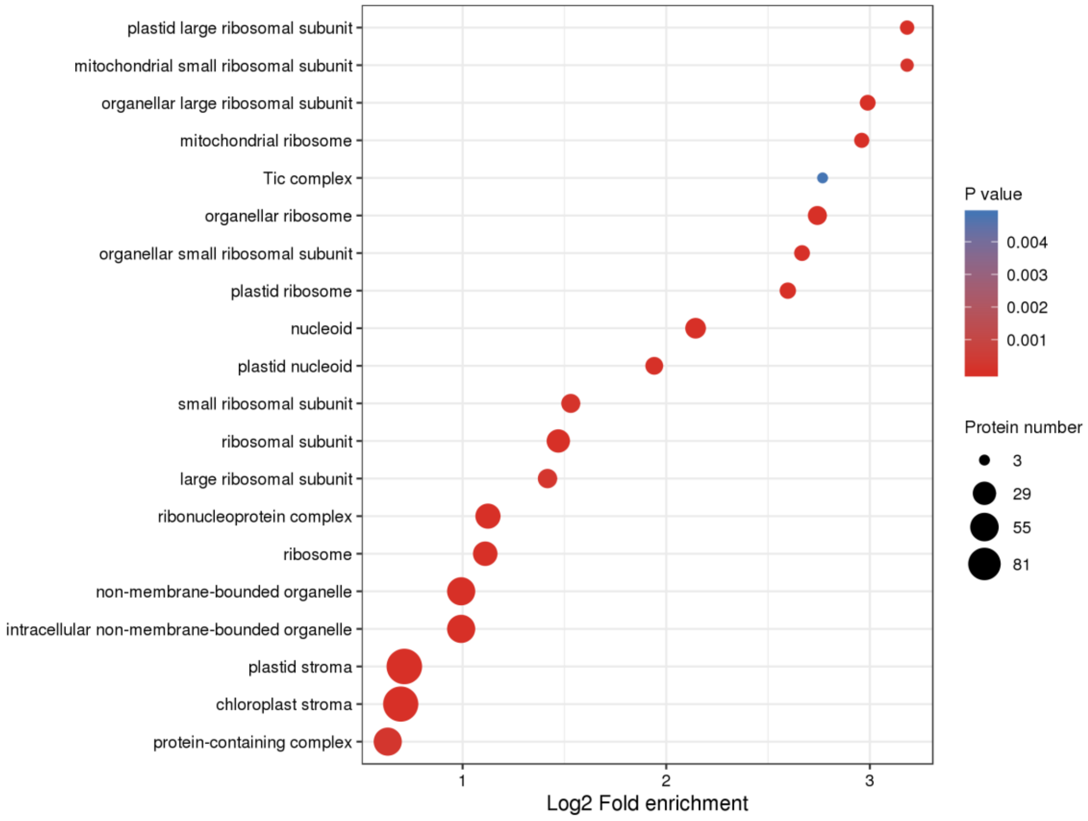


**Fig. S5. Go analysis of proteins up-regulated in *tsl2* mutant plants at 18ºC.**

Criteria for differential expression were set as q ≤ 0.05, fold change (FC) ≥1.3 for upregulation. Totally 208 up-regulated proteins in *tsl2* mutant at 18ºC were used for GO analysis.
